# Exploring the Relationship Between Obstructive Sleep Apnea and Olfactory Function

**DOI:** 10.3390/life15040675

**Published:** 2025-04-21

**Authors:** Antonino Maniaci, Mario Lentini, Maria Rita Bianco, Daniele Salvatore Paternò, Salvatore Lavalle, Annalisa Pace, Giannicola Iannella, Paolo Boscolo-Rizzo, Miguel Mayo-Yanez, Christian Calvo-Henriquez, Jerome R. Lechien, Luigi La Via

**Affiliations:** 1 Department of Medicine and Surgery, University of Enna “Kore”, 94100 Enna, Italy; mario.lentini@unikore.it (M.L.); salvatore.lavalle@unikore.it (S.L.); 2 ASP Ragusa, 97100 Ragusa, Italy; paternomd@icloud.com; 3 Study Group of Young-Otolaryngologists of the International Federations of Oto-Rhino-Laryngological Societies (YO-IFOS), 75019 Paris, France; giannicola.iannella@uniroma1.it (G.I.); miguelmmy@gmail.com (M.M.-Y.); christian.calvo.henriquez@gmail.com (C.C.-H.); jerome.lechien@umons.ac.be (J.R.L.); 4 Otolaryngology Unit, Department of Health Science, University Magna Graecia of Catanzaro, Viale Europa, Germaneto, 88100 Catanzaro, Italy; mrbianco@unicz.it; 5 Department of ‘Organi di Senso’, University “Sapienza”, 00185 Rome, Italy; annalisa.pace@uniroma1.it; 6 Section of Otolaryngology, Department of Medical, Surgical and Health Sciences, University of Trieste, 34127 Trieste, Italy; paolo.boscolorizzo@units.it; 7 Otorhinolaryngology, Head and Neck Surgery Department, Complexo Hospitalario Universitario A Coruña (CHUAC), 15006 A Coruña, Spain; 8 Service of Otolaryngology, Hospital Complex of Santiago de Compostela, 15706 Santiago de Compostela, Spain; 9 Department of Anatomy and Experimental Oncology, UMONS Research Institute for Health Sciences and Technology, Mons School of Medicine, University of Mons (UMons), 7011 Mons, Belgium; 10 Department of Anesthesia and Intensive Care 1, University Hospital Policlinico “G. Rodolico-San Marco”, 95123 Catania, Italy; luigilavia7@gmail.com

**Keywords:** obstructive sleep apnea, olfactory dysfunction, chronic intermittent hypoxia, Inflammation, olfactory assessment, continuous positive airway pressure, neurocognitive function, sleep fragmentation, olfactory training, multidisciplinary management

## Abstract

Obstructive sleep apnea (OSA) is increasingly recognized as a chronic condition that is closely interrelated to olfactory disorders, with a significant contribution to quality of health and overall quality of life. This narrative review aims to provide a thorough overview of the emerging evidence that now integrates these two previously considered distinct physiologic systems. Studies published recently have reported a significantly higher frequency of olfactory dysfunction among OSA patients compared to the general population, which raises the possibility of a causal relationship. We explore the postulated mechanisms behind this association, namely, the chronic intermittent hypoxia, local inflammatory effect, and neuroanatomical changes attributed to OSA. The review further explores the clinical impacts of this relationship through proposing the potential for an olfactory assessment to be used as a diagnostic modality for OSA and the effects of OSA treatment on olfactory function. Thus, we explore the difficulties in treating patients who experience both and suggest future areas for research. This review attempts to bridge the gap between the existing literature and impending investigation necessary for a better management of the interaction of sleep apnea and the human sense of smell.

## 1. Introduction

Sleep apnea and olfactory disorders are two distinct medical conditions that have gained increasing attention in recent years, given their considerable impact on public health and quality of life. Although these conditions appear to be unrelated at first glance, recent evidence indicates interesting associations between them, highlighting the need for an overview of their relationship and possible common mechanisms. Sleep apnea syndrome is one of the most common sleep disorders, defined by the repeated occurrence of partial (hypopnea, reduced airflow) or complete (apnea) upper airway obstruction during sleep resulting in recurrent episodes of oxygen desaturation and sleep fragmentation [1]. Sleep apnea is a major cause of morbidity and mortality worldwide and recent estimates indicate that around 1 billion adults suffer from sleep apnea [2]. This increase in prevalence has, in part, been ascribed to the current obesity epidemic, as well as the ageing populations in many countries [3]. Untreated sleep apnea has profound effects on many organ systems, including the cardiovascular, metabolic, and neurocognitive systems, which collectively affect health care systems and quality of life for patients [4]. Olfactory disorders, on the other hand, are a heterogeneous group of diseases characterized by an alteration of the sense of smell (a partial or complete loss of olfactory function (hyposmia/anosmia)) [5]. The importance of olfaction in everyday life is often underestimated. Olfaction is critical for flavor perception, food pleasure, social behavior, and danger awareness in the surrounding environment [6]. Recent events worldwide, namely, the COVID-19 pandemic, have pushed olfactory disorders to the foreground, as anosmia became a range of key features of SARS-CoV-2 infection [7]. Such awareness had led to increased research focused on the mechanisms of olfactory dysfunction and potential therapeutic options. As of recently, the scientific evidence of a possible link between sleep apnea and olfactory disorders has only begun to emerge. Previous studies have found a markedly elevated prevalence of olfactory dysfunction in individuals with obstructive sleep apnea (OSA) relative to community norms [8,9]. This association raises important questions regarding the underlying mechanisms linking these two conditions and the effect of sleep apnea treatment on olfactory functioning. Serious candidate mechanisms linking olfactory dysfunction and sleep apnea suggest the effects of chronic intermittent hypoxia on both the olfactory epithelium and central olfactory processing pathways [10]. Nasal mucosa exposure to the cyclic reoxygenation and oxygen desaturation found in sleep apnea can cause oxidative stress and inflammation, which can then attack and kill olfactory sensory neurons [11]. Moreover, the systemic inflammation induced by sleep apnea may influence the olfactory bulb and secondary olfactory processing sites within the brain [12]. An interesting alternative hypothesis proposes that the repeated collapse of the airway seen in sleep apnea could lead to upper airway inflammation and edema, which directly affects the nasal mucosa and olfactory epithelium [13]. Such localized inflammation may change the microenvironment of olfactory receptor cells [14], consequently hindering odor detection and discrimination. Additionally, many neurocognitive sequelae of OSA, such as changes in the structure and function of the brain, might indirectly compromise olfactory processing [15]. Recent neuroimaging studies revealed the alteration of gray matter volume and functional connectivity of brain areas involved in sleep regulation and olfaction processing in patients with obstructive sleep apnea [16]. It is also important to consider the bidirectionality of this relationship. Olfactory dysfunction can also play a role in the pathogenesis or worsening of sleep apnea owing to its effects on upper airway muscle tone and respiratory control [17]. Impaired olfactory function may also modulate respiratory patterns and arousal responses, implicating its potential role in sleep-disordered breathing [18].

Another common reason would be the limitation of airflow, which is an important anatomical and functional component that overlaps both OSA and olfactory dysfunction, best known as nasal obstruction. In the case of OSA, nasal resistance is an important factor in upper airway collapsibility, especially in patients with a deviated septum, turbinate hypertrophy, and chronic rhinosinusitis. Nasal blockage may also limit odorant access to the olfactory epithelium, causing a reduced perception of odorants (conductive olfactory loss). It is also of note that, in both allergic rhinitis and sinonasal disease, chronic nasal inflammation can lead to epithelial remodeling and olfactory receptor neuron damage, further exacerbating the impact of the intermittent hypoxia mediated by nasal obstruction. Thus, it can be concluded that ventilatory impairment must be addressed in the comprehensive management of patients with OSA and olfactory impairment. In addition, nasal obstruction is a frequent contributing factor to both olfactory dysfunction and OSA. Patients compensate by mouth breathing (which bypasses the olfactory epithelium and decreases odorant detection) when nasal air flow decreases, and this leads to the occurrence of hyposmia or anosmia. Mouth breathing changes upper airway dynamics as well, favoring a lower tongue posture and decreased oropharyngeal muscle tone in sleep, both conditions associated with an increased risk of snoring with pharyngeal collapse—both markers of obstructive sleep apnea. Nasal resistance is also positively correlated with the apnea–hypopnea index (AHI), especially in patients with allergic rhinitis, a nasal septum deviation, and chronic nasal mucosal inflammation [11,12]. Accordingly, nasal obstruction may serve as a common pathway connecting dysfunctional olfaction and sleep-disordered breathing.

The identification potential spectrum of olfactory disorder in sleep apnea has important clinical implications. A specific example might be screening for olfactory dysfunction in sleep apnea patients, allowing for the detection of those at risk for neurodegenerative diseases, as olfactory dysfunction is often an early condition in patients with diseases such as Alzheimer’s and Parkinson’s [19]. Moreover, exploring the effects of sleep apnea therapy on olfactory dysfunction may give insight into the reversibility of olfactory deficits and help design therapeutic strategies for both conditions [20]. This thorough review intends to integrate the existing literature on sleep apnea and olfactory disorders, examine the potential mechanisms linking these two conditions, and, ultimately, discuss the clinical significance of their association. We aim to prospectively analyze the existing literature and highlight unanswered questions to encourage research, and, ultimately, improve patient outcomes, in this evolving area. The complex interrelationships between the two conditions presented here necessitate a coordinated, multidisciplinary approach including input from sleep medicine, otolaryngology, neurology, and basic science to fully understand this relationship. The results described in this review may not only enhance our understanding of these three diseases but also allow us to define new therapeutic strategies and provide better clinical results for the patients.

## 2. Materials and Methods

This narrative review was conducted to synthesize and analyze the current literature on the relationship between sleep apnea and olfactory disorders. The methodology employed in this review process is described below. A comprehensive search of electronic databases was performed, including PubMed, MEDLINE, Embase, and the Cochrane Library. The literature search aimed to capture both foundational and recent developments in the field, including emerging insights into the impact of COVID-19 on olfactory function and its potential overlap with sleep-disordered breathing. No strict publication date restrictions were applied, allowing the inclusion of both classic references and the most up-to-date findings.

The search terms included combinations and variations of keywords related to sleep apnea, olfactory disorders, and their potential interactions. Additional relevant articles were identified through manual searching of reference lists from key papers and reviews. Studies were included if they met the following criteria: (1) published in English, (2) focused on sleep apnea and/or olfactory disorders in human subjects, (3) investigated the relationship between sleep apnea and olfactory function, or (4) examined the mechanisms potentially linking these conditions. Exclusion criteria included studies solely focused on animal models, case reports, and articles that were not peer-reviewed. Relevant data from the included studies were extracted by two independent authors and summarized. This included information on study design, sample size, methodologies used, main findings, and conclusions. The extracted data were then analyzed to identify common themes, conflicting results, and gaps in current knowledge. The findings from this comprehensive review process form the basis of the subsequent sections, providing a critical analysis of the current state of knowledge regarding the relationship between sleep apnea and olfactory disorders.

## 3. What Do We Know About Sleep Apnea?

**Figure 1:**
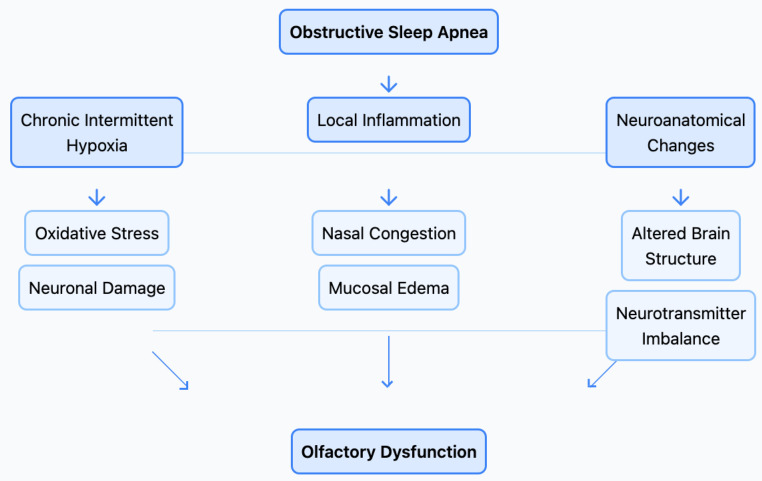
Tree-style flowchart depicting the interrelated mechanisms through which obstructive sleep apnea (OSA) contributes to olfactory dysfunction. The diagram categorizes the key pathways—chronic intermittent hypoxia, local inflammation, and neuroanatomical changes—highlighting their downstream effects, such as oxidative stress, neuronal damage, nasal congestion, and neurotransmitter imbalances, which collectively impair olfactory function.

Sleep apnea is a complex sleep disorder characterized by recurrent episodes of complete or partial upper airway obstruction during sleep, leading to intermittent hypoxia, hypercapnia, and sleep fragmentation [21]. The three main types of sleep apnea are OSA, central sleep apnea (CSA), and complex sleep apnea syndrome (CompSAS), with OSA being the most prevalent form [22]. The pathophysiology of OSA involves a multifaceted interplay between anatomical and neuromuscular factors. Anatomically, a narrow upper airway predisposes individuals to airway collapse during sleep [23]. This narrowing can result from factors such as obesity, craniofacial abnormalities, or enlarged tonsils and adenoids in children [24]. Neuromuscular factors play a crucial role in maintaining upper airway patency during sleep. In OSA patients, there is often impaired neuromuscular compensation, leading to the insufficient activation of upper airway dilator muscles during inspiratory effort [25] (Figure 1).

**Figure 2:**
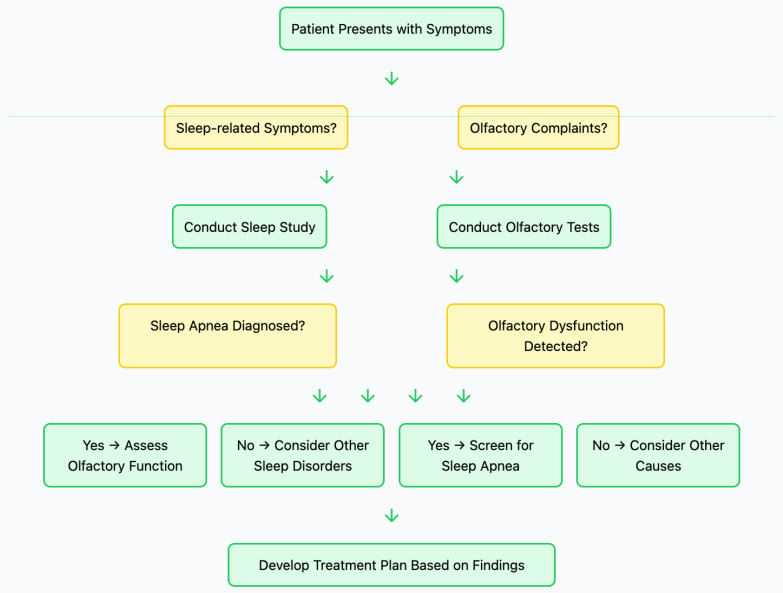
Flowchart illustrating the proposed pathophysiological mechanisms linking obstructive sleep apnea (OSA) to olfactory dysfunction. The diagram highlights three primary pathways, chronic intermittent hypoxia, local inflammation, and neuroanatomical changes, each contributing to neuronal damage, oxidative stress, mucosal edema, and neurotransmitter imbalances, ultimately leading to impaired olfactory function.

Recent research has shed light on the role of loop gain in OSA pathogenesis. Loop gain, a measure of the stability of the respiratory control system, has been found to be elevated in many OSA patients, contributing to breathing instability during sleep [26]. Additionally, the arousal threshold, which determines an individual’s propensity to wake up in response to respiratory stimuli, plays a significant role in OSA severity. A low arousal threshold can lead to frequent arousals and perpetuate breathing instability [27]. The risk factors for OSA are numerous and often interrelated. Obesity remains the most significant modifiable risk factor, with a 10% weight gain associated with a 32% increase in the apnea–hypopnea index (AHI) [28]. Age is another crucial factor, with OSA prevalence increasing in older adults due to changes in upper airway collapsibility and respiratory control stability [29]. The male sex is also a risk factor, although the gender gap narrows after menopause, suggesting a protective role of female sex hormones [30]. Genetic factors contribute to OSA risk, with studies estimating heritability between 30–40% [31]. Recent genome-wide association studies have identified several genetic loci associated with OSA, including genes involved in craniofacial development, body fat distribution, and sleep–wake regulation [32]. The recognition and diagnosis of OSA have evolved significantly in recent years. While snoring and witnessed apneas remain important clinical indicators, the heterogeneity of OSA presentations is now better appreciated. Symptoms such as excessive daytime sleepiness, morning headaches, and mood disturbances are recognized as potential manifestations of OSA [33]. However, it is important to note that many OSA patients, particularly women and older adults, may not present with classic symptoms, underscoring the need for increased awareness and screening [34]. Diagnostic approaches have also advanced, moving beyond traditional in-laboratory polysomnography (PSG) (Figure 2).

Home sleep apnea testing (HSAT) has gained acceptance as a cost-effective alternative for diagnosing OSA in selected patients [35]. Novel technologies, such as peripheral arterial tonometry and mandibular movement monitoring, offer promising alternatives for OSA screening and diagnosis [36]. Treatment options for OSA have expanded in recent years, with continuous positive airway pressure (CPAP) remaining the gold standard for moderate to severe OSA [37]. However, adherence to CPAP remains a significant challenge, with studies reporting long-term adherence rates of only 30–60% [38]. This has led to increased interest in alternative treatments. Oral appliances, particularly mandibular advancement devices, have shown efficacy in mild to moderate OSA and in CPAP-intolerant patients [39]. Positional therapy, which aims to prevent supine sleep, has also demonstrated effectiveness in positional OSA [40]. Emerging therapies include hypoglossal nerve stimulation, which has shown promising results in selected patients with moderate to severe OSA [41]. Surgical interventions for OSA have evolved from traditional uvulopalatopharyngoplasty to more targeted approaches. Maxillomandibular advancement surgery has demonstrated high success rates in carefully selected patients [42]. Newer techniques, such as drug-induced sleep endoscopy, allow for better surgical planning by identifying specific sites of obstruction during sleep [43]. The long-term consequences of untreated OSA are substantial and extend beyond sleep-related symptoms. Cardiovascular complications, including hypertension, coronary artery disease, and stroke, are well-documented sequelae of OSA [44]. Metabolic disturbances, particularly insulin resistance and type 2 diabetes, are also strongly associated with OSA [45]. Neurocognitive impairment, including deficits in attention, executive function, and memory, can significantly impact daily functioning and quality of life in OSA patients [46]. Recent research has also highlighted the bidirectional relationship between OSA and various comorbidities. For instance, OSA has been implicated in the pathogenesis and progression of chronic kidney disease [47]. Conversely, conditions such as heart failure and chronic opioid use can exacerbate or even induce central sleep apnea [48]. As our understanding of sleep apnea continues to evolve, so too does our approach to management. Personalized treatment strategies, based on individual phenotypes and endotypes, are emerging as the future of OSA care [49]. By considering factors such as upper airway anatomy, loop gain, and arousal threshold, clinicians may be able to tailor treatments more effectively to individual patients, potentially improving outcomes and adherence [50].

## 4. How Do Olfactory Disorders Affect Human Health?

The olfactory system plays a crucial role in human health and well-being, extending far beyond the simple perception of odors. Understanding its structure, function, and disorders is essential for appreciating the full impact of olfactory dysfunction on human health. The olfactory system consists of the olfactory epithelium, olfactory bulb, and higher-order olfactory processing regions in the brain [51]. Odorant molecules bind to olfactory receptors in the olfactory epithelium, triggering a cascade of events that, ultimately, results in odor perception. Recent research has revealed the complexity of this system, with humans possessing approximately 400 functional olfactory receptor genes, capable of detecting thousands of distinct odors [52,53]. Olfactory dysfunction can manifest in various forms, including hyposmia (reduced smell sensitivity), anosmia (the complete loss of smell), parosmia (distorted smell perception), and phantosmia (the perception of non-existent odors) [5]. These disorders can be classified as quantitative (affecting odor threshold) or qualitative (altering odor quality perception) [54]. The prevalence of olfactory disorders increases with age, affecting up to 25% of individuals over 53 years old [54]. The etiology of olfactory disorders is diverse, encompassing congenital, post-traumatic, post-viral, neurodegenerative, and idiopathic causes [55]. Viral upper respiratory infections, including COVID-19, have emerged as a leading cause of sudden-onset olfactory dysfunction [56]. Post-traumatic olfactory loss, often resulting from head injuries, can have long-lasting effects due to the vulnerability of olfactory nerve fibers passing through the cribriform plate [57]. Neurodegenerative diseases, particularly Parkinson’s and Alzheimer’s, are increasingly recognized for their association with olfactory dysfunction. Olfactory impairment often precedes motor and cognitive symptoms in these conditions, potentially serving as an early biomarker [58]. Recent research has also highlighted the role of air pollution in olfactory dysfunction, with exposure to particulate matter and other pollutants linked to reduced olfactory function [59]. Diagnosing and assessing olfactory disorders require a multifaceted approach. Psychophysical tests, such as the University of Pennsylvania Smell Identification Test (UPSIT) and Sniffin’ Sticks, remain the gold standard for quantifying olfactory function [60]. These tests evaluate odor identification, discrimination, and threshold detection. Newer techniques, including olfactory event-related potentials and functional magnetic resonance imaging, offer insights into the neural correlates of olfactory processing [61]. Electro-olfactography, a technique measuring electrical responses in the olfactory epithelium, has shown promise in objectively assessing peripheral olfactory function [62]. Additionally, recent advances in machine learning have led to the development of automated olfactory testing methods, potentially improving the efficiency and accuracy of diagnosis [63]. Treatment options for olfactory disorders have traditionally been limited, but recent research has yielded promising approaches. Olfactory training, involving repeated exposure to specific odors, has demonstrated efficacy in improving olfactory function across various etiologies [64]. This technique is thought to stimulate neuroplasticity in the olfactory system, promoting the regeneration and reorganization of neural pathways [65]. Pharmacological interventions, while still limited, show potential in certain cases. Corticosteroids have shown efficacy in treating some forms of post-viral olfactory loss, particularly when administered early in the course of the disorder [66]. Emerging research into the use of growth factors and stem cell therapies offers hope for more targeted treatments in the future [67]. The impact of olfactory disorders on quality of life is profound and often underestimated. Olfaction plays a crucial role in flavor perception, with up to 80% of what we perceive as taste actually stemming from smell [68]. Consequently, olfactory dysfunction can significantly affect food enjoyment and nutritional intake, potentially leading to weight loss and malnutrition, particularly in older adults [69]. Beyond nutrition, olfactory disorders can compromise safety, as affected individuals may struggle to detect warning odors such as smoke, gas leaks, or spoiled food [70]. This can lead to increased anxiety and social isolation, as individuals become hyper-vigilant about potential hazards or embarrassed about their condition [71]. The emotional and social ramifications of olfactory dysfunction are substantial. Smell is intimately linked to memory and emotion, with olfactory impairment potentially affecting emotional processing and well-being [72]. Recent studies have reported higher rates of depression and anxiety in individuals with olfactory disorders, highlighting the need for psychological support in management [73]. Occupational impacts can be significant, particularly in professions relying heavily on olfactory function, such as chefs, sommeliers, and perfumers [74]. The COVID-19 pandemic has brought increased attention to these occupational challenges, as sudden-onset anosmia has affected workers across various industries [75]. As our understanding of olfactory disorders grows, so does the recognition of their far-reaching impact on human health. Future research directions include developing more targeted therapies based on specific olfactory disorder phenotypes, exploring the potential of olfactory dysfunction as a biomarker for neurodegenerative diseases, and investigating the long-term outcomes of COVID-19-related olfactory loss [76,77].

## 5. How Common Are Olfactory Disorders in Sleep Apnea Patients?

The relationship between sleep apnea and olfactory function has emerged as an intriguing area of research, with growing evidence suggesting a significant connection between these two seemingly disparate conditions. Understanding this relationship could have important implications for both the diagnosis and treatment of these disorders. Recent studies have indicated that olfactory disorders are more prevalent in sleep apnea patients compared to the general population. A cross-sectional study by Salihoglu et al. found that 30% of patients with OSA had hyposmia, compared to only 3% in the control group [8]. Similarly, a significant negative correlation between the severity of OSA, as measured by the apnea–hypopnea index (AHI), and olfactory function scores was reported [78,79]. These findings suggest that olfactory dysfunction may be a common, yet often overlooked, comorbidity in sleep apnea patients. The mechanisms linking sleep apnea and olfactory disorders are multifaceted and not fully elucidated. However, several hypotheses have been proposed to explain this connection. One primary mechanism involves the effects of chronic intermittent hypoxia (CIH), a hallmark of sleep apnea, on the olfactory system. CIH has been shown to induce oxidative stress and inflammation in various tissues, including the nasal mucosa and olfactory epithelium [80,81]. A study by Nacher-Carda et al. demonstrated that OSA patients had increased levels of inflammatory markers in their nasal mucosa, which correlated with decreased olfactory function [11]. Furthermore, the repeated episodes of upper airway collapse in OSA may lead to mechanical trauma and edema in the nasal and pharyngeal tissues. This local inflammation could directly impact the olfactory epithelium and impair odor detection [82,83]. Another potential mechanism involves the impact of sleep fragmentation, another key feature of sleep apnea, on olfactory processing. Sleep plays a crucial role in memory consolidation, including olfactory memory. Disrupted sleep patterns in OSA patients may interfere with this process, potentially leading to impaired olfactory function [84]. A recent study found that OSA patients had a reduced gray matter volume in brain regions associated with olfactory processing, suggesting a potential neuroanatomical basis for olfactory dysfunction in these patients [85]. The bidirectional nature of this relationship should also be considered. Olfactory dysfunction may contribute to the pathogenesis or exacerbation of sleep apnea through its effects on upper airway muscle tone and respiratory control. The olfactory system has connections to brainstem regions involved in respiratory regulation, and impaired olfactory input could potentially alter these regulatory mechanisms [86]. Additionally, reduced olfactory function may lead to changes in eating behaviors and weight gain, a known risk factor for OSA [87]. Intriguingly, emerging evidence suggests that treating sleep apnea may lead to improvements in olfactory function. A prospective study by Walliczek-Dworschak et al. found that CPAP therapy, the gold standard treatment for OSA, resulted in significant improvements in olfactory function after three months of treatment [14]. These improvements were correlated with reductions in AHI and daytime sleepiness. Similarly, a study by Cavaliere et al. demonstrated that the surgical treatment of OSA through uvulopalatopharyngoplasty led to significant improvements in olfactory function, particularly in odor identification and discrimination [88]. These findings suggest that addressing the underlying sleep apnea may have beneficial effects on olfactory performance. The potential for olfactory dysfunction to serve as a biomarker for sleep apnea is an exciting area of research. Given the high prevalence of undiagnosed OSA and the challenges associated with traditional diagnostic methods, identifying easily assessable markers could greatly improve screening and diagnosis. A recent pilot study by Jiang et al. found that a combination of olfactory function tests and questionnaires could predict the presence of OSA with a high sensitivity and specificity [89]. However, it is important to note that the relationship between sleep apnea and olfactory function is complex and likely influenced by various factors, including age, BMI, and comorbidities. A large-scale, longitudinal study found that, while OSA was associated with an increased risk of olfactory dysfunction, this relationship was attenuated after adjusting for these confounding factors [90]. This highlights the need for the careful consideration of multiple variables in future research. As our understanding of the connection between sleep apnea and olfactory function continues to evolve, several key areas warrant further investigation. These include the long-term effects of OSA on olfactory function, the potential for olfactory training as an adjunct therapy in OSA patients, and the role of genetic factors in mediating this relationship [91]. Additionally, exploring the impact of novel OSA treatments, such as hypoglossal nerve stimulation, on olfactory outcomes could provide valuable insights [92,93].

## 6. Clinical Implications

**Figure 3:**
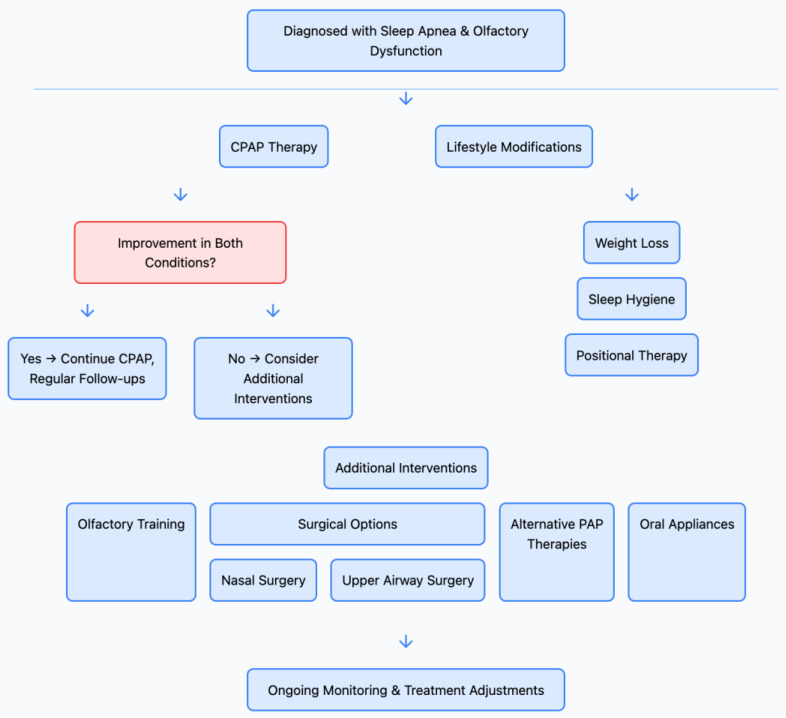
Diagnostic decision tree outlining the approach to evaluating patients presenting with sleep-related and olfactory symptoms. The flowchart demonstrates the sequential assessment process, including sleep studies, olfactory function tests, and differential diagnosis considerations, leading to the development of an appropriate treatment plan based on clinical findings.

The emerging relationship between sleep apnea and olfactory disorders carries significant clinical implications, prompting a re-evaluation of current diagnostic and treatment approaches. As our understanding of this connection deepens, it becomes increasingly important to consider how this knowledge can be translated into improved patient care. The question of whether to screen for olfactory disorders in sleep apnea patients is gaining traction in the medical community. Given the higher prevalence of olfactory dysfunction in this population, routine screening could potentially identify a significant number of cases that might otherwise go undetected. Nearly 40% of OSA patients had some degree of olfactory impairment, suggesting that a substantial portion of these patients may benefit from early detection and intervention [94]. Implementing olfactory screening in sleep clinics could serve multiple purposes. Firstly, it could help identify patients at risk for complications associated with olfactory dysfunction, such as nutritional deficiencies or safety hazards [95]. Secondly, changes in olfactory function could potentially serve as a marker for OSA progression or treatment efficacy. A longitudinal study demonstrated that improvements in olfactory function were correlated with successful CPAP therapy, suggesting that olfactory testing could be a useful tool in monitoring treatment response [96]. However, the decision to implement widespread screening must be balanced against practical considerations. Olfactory testing requires specialized equipment and training, which may not be readily available in all sleep clinics. Additionally, the cost-effectiveness of such screening needs to be evaluated. A recent cost–utility analysis by Patel et al. suggested that, while olfactory screening in OSA patients could be beneficial, its cost-effectiveness may depend on factors such as test accuracy and the availability of effective interventions for olfactory dysfunction [97]. If screening is implemented, the choice of testing method is crucial. While comprehensive tests like the University of Pennsylvania Smell Identification Test (UPSIT) provide detailed information, they can be time-consuming and expensive. Shorter screening tools, such as the Sniffin’ Sticks 12-item test, may offer a more practical alternative for initial screening in sleep clinics [98]. Recent advancements in digital olfactory testing, such as the development of smartphone-based applications, could potentially make screening more accessible and cost-effective [99]. The management of patients with both sleep apnea and olfactory disorders presents unique challenges and opportunities. A multidisciplinary approach, involving sleep specialists, otolaryngologists, and neurologists, is likely to yield the best outcomes. The primary goal should be to address both conditions concurrently, as evidence suggests that treating one may have beneficial effects on the other. For OSA patients with olfactory dysfunction, optimizing CPAP therapy should be a priority. In addition to core specialties such as Neurology, Sleep Medicine, and Otolaryngology, other adjunctive interventions, as represented in Figure 3, should be considered (Figure 3). Cognitive-behavioral therapy, respiratory physiotherapy, and lifestyle modification may also be undertaken depending on unique patient clinical features. These adjunctive strategies may be especially important in those with substantive neurocognitive compromise, chronic nasal obstruction, or poor CPAP tolerance. Translating to patient-centered care is the way the diagnosis is obtained in the figure, as well as holistic and patient-tailored care including symptom management, functional recovery, and behavior; and, therefore, these are included in the figure.

A recent study found that CPAP adherence was associated with greater improvements in olfactory function, highlighting the importance of patient education and support to enhance treatment compliance [100]. Additionally, considering alternative OSA treatments, such as mandibular advancement devices or upper airway surgery, may be beneficial for patients who cannot tolerate CPAP and have concurrent olfactory issues [101]. Specific interventions for olfactory dysfunction should also be integrated into the management plan. Olfactory training, which involves the repeated exposure to specific odors, has shown promise in improving olfactory function across various etiologies [64]. A randomized controlled trial demonstrated that olfactory training was effective in improving olfactory function in patients with post-infectious olfactory loss, and similar benefits might be observed in OSA patients [102]. Addressing nasal inflammation, which may contribute to both OSA and olfactory dysfunction, is another important aspect of management. Topical corticosteroids have shown efficacy in improving nasal patency and olfactory function in patients with chronic rhinosinusitis, and their use could be considered in OSA patients with concomitant nasal inflammation [103]. However, the long-term safety and efficacy of these treatments in the context of OSA need further investigation. Nutritional counseling should also be considered for patients with both conditions. Olfactory dysfunction can lead to altered eating habits and potential nutritional deficiencies, which may exacerbate OSA symptoms. A study by Bhattacharyya et al. found that patients with olfactory dysfunction had significantly lower serum zinc levels, suggesting that micronutrient supplementation may be beneficial in some cases [104]. The regular follow-up and reassessment of both olfactory function and sleep apnea severity are crucial. Changes in olfactory function could potentially signal a worsening of OSA or the need for treatment adjustments. Conversely, improvements in olfactory function following OSA treatment could serve as a motivating factor for patients to adhere to their therapy [105]. Future research should focus on developing standardized protocols for screening and managing patients with both conditions. Large-scale, longitudinal studies are needed to better understand the long-term outcomes of various management strategies. Additionally, investigating the potential of novel therapies, such as neuromodulation techniques or targeted anti-inflammatory treatments, could open up new avenues for managing this complex patient population [106]. As our understanding of this relationship continues to evolve, so too will our ability to provide optimal care for these patients, potentially improving both their sleep quality and their overall quality of life [107].

## 7. Future Research

As our understanding of the relationship between sleep apnea and olfactory disorders continues to evolve, it becomes increasingly clear that there are still many unanswered questions and areas ripe for further investigation. This final section explores the current gaps in our knowledge and proposes directions for future research that could significantly advance our understanding and management of these interrelated conditions. One of the primary areas of uncertainty lies in the exact mechanisms linking sleep apnea and olfactory dysfunction. While several hypotheses have been proposed, including the effects of chronic intermittent hypoxia and local inflammation, the precise pathophysiological pathways remain to be fully elucidated [108]. Future studies should employ advanced imaging techniques, such as functional MRI and PET scans, to better understand the neuroanatomical and functional changes in the olfactory system of sleep apnea patients [109]. The temporal relationship between the onset of sleep apnea and olfactory dysfunction is another area that requires clarification. Longitudinal studies tracking olfactory function in individuals at risk for sleep apnea could provide valuable insights into whether olfactory changes precede or follow the development of sleep-disordered breathing [20,110]. Such information could potentially lead to the use of olfactory testing as an early screening tool for sleep apnea. The role of genetic factors in mediating the relationship between sleep apnea and olfactory function is an intriguing area for future research. Recent advances in genomics have identified several genes associated with both conditions independently, but their potential overlap and interaction remain largely unexplored [91]. Genome-wide association studies focusing on patients with both sleep apnea and olfactory dysfunction could uncover novel genetic markers and potential therapeutic targets. Another critical area for investigation is the long-term impact of sleep apnea treatment on olfactory function. While some studies have shown improvements in olfactory performance following CPAP therapy, the durability of these effects and their relationship to treatment adherence are not well-understood [55]. Long-term follow-up studies comparing different treatment modalities (e.g., CPAP, oral appliances, and surgery) and their effects on olfactory function could provide valuable guidance for clinical decision-making. The potential use of olfactory function as a biomarker for sleep apnea severity and treatment response is an exciting prospect that warrants further exploration. Developing standardized protocols for olfactory testing in sleep clinics and correlating these results with polysomnographic data could lead to more efficient monitoring and personalized treatment strategies [111]. The impact of comorbidities on the relationship between sleep apnea and olfactory function is another area that requires further investigation. Conditions such as diabetes, hypertension, and neurodegenerative diseases are known to affect both sleep and olfaction independently, but their combined effects in the context of sleep apnea are not well-understood [112]. Future studies should aim to disentangle these complex interactions and their implications for patient management. The potential role of olfactory training in improving outcomes for patients with both sleep apnea and olfactory dysfunction is an intriguing area for future research. While olfactory training has shown promise in various olfactory disorders, its efficacy in the context of sleep apnea has not been thoroughly investigated [113]. Randomized controlled trials evaluating the effects of olfactory training as an adjunct to standard sleep apnea treatments could potentially lead to novel therapeutic approaches. The development of novel therapeutic interventions targeting both sleep apnea and olfactory dysfunction simultaneously is an exciting frontier for future research. For instance, exploring the potential of neuromodulation techniques, such as transcranial magnetic stimulation or vagus nerve stimulation, in addressing both conditions could open up new avenues for treatment [114]. Investigating the impact of environmental factors, particularly air pollution, on the relationship between sleep apnea and olfactory function is another important area for future research. With increasing evidence linking air pollution to both sleep-disordered breathing and olfactory impairment, understanding these interactions could have significant public health implications [115]. The potential role of the microbiome in mediating the relationship between sleep apnea and olfactory function is an emerging area of interest. Recent studies have shown that both conditions are associated with alterations in the nasal and gut microbiome, but the implications of these changes and their potential as therapeutic targets remain to be explored [116]. Future research should also focus on developing more sensitive and specific diagnostic tools for both sleep apnea and olfactory disorders. This could include the development of point-of-care devices for rapid olfactory testing or advanced wearable technologies for home-based sleep apnea diagnosis [117]. The economic impact of concurrent sleep apnea and olfactory dysfunction, including healthcare utilization and productivity losses, is an important area for future investigation. Cost-effectiveness analyses of various screening and treatment strategies could provide valuable guidance for healthcare policy and resource allocation [118]. Lastly, there is a need for more diverse and representative study populations in future research. Many existing studies have been limited by small sample sizes or homogeneous populations. Large-scale, multicenter studies including diverse ethnic and socioeconomic groups are necessary in order to ensure the generalizability of the findings and to identify any population-specific factors influencing the relationship between sleep apnea and olfactory function [119].

## 8. Conclusions

The relationship between sleep apnea and olfactory disorders represents a fascinating intersection of two seemingly distinct physiological systems. This review has highlighted the growing evidence for a significant connection between these conditions, with implications for both patient care and scientific understanding. The higher prevalence of olfactory dysfunction in sleep apnea patients, coupled with the potential improvements in olfactory function following sleep apnea treatment, underscores the importance of considering these conditions in tandem. The proposed mechanisms linking the two, including chronic intermittent hypoxia, local inflammation, and neuroanatomical changes, offer intriguing avenues for further research and potential therapeutic interventions. From a clinical perspective, the findings presented here suggest that olfactory assessment could become a valuable tool in the diagnosis and management of sleep apnea. Conversely, sleep evaluation may be warranted in patients presenting with unexplained olfactory dysfunction. This bidirectional approach could lead to earlier detection and more comprehensive treatment strategies for both conditions. However, numerous questions remain unanswered, particularly regarding the exact nature of the causal relationship between sleep apnea and olfactory disorders, the long-term outcomes of various treatment approaches, and the potential for olfactory function to serve as a biomarker for sleep apnea severity or treatment efficacy. As research in this field progresses, it is likely to yield valuable insights that could revolutionize our approach to both sleep and olfactory disorders. The potential for integrated treatment strategies that address both conditions simultaneously is particularly exciting and could significantly improve patient outcomes and quality of life. Ultimately, this emerging field of study serves as a powerful reminder of the interconnected nature of human physiology and the importance of holistic approaches in medical research and practice. As we continue to explore the complex relationship between sleep apnea and olfactory function, we move closer to a more comprehensive understanding of these conditions and, potentially, to more effective ways of treating them.

## Data Availability

Not applicable.

## Abbreviations

The following abbreviations are used in this manuscript:

AHI	Apnea–Hypopnea Index
CIH	Chronic Intermittent Hypoxia
CompSAS	Complex Sleep Apnea Syndrome
COPD	Chronic Obstructive Pulmonary Disease
COVID-19	Coronavirus Disease 2019
CPAP	Continuous Positive Airway Pressure
CRS	Chronic Rhinosinusitis
CSA	Central Sleep Apnea
HSAT	Home Sleep Apnea Testing
MOIT	Monell Olfactory Image Test
OSA	Obstructive Sleep Apnea
PSG	Polysomnography
SARS-CoV-2	Severe Acute Respiratory Syndrome Coronavirus 2
UPSIT	University of Pennsylvania Smell Identification Test
